# Status of alternative angiogenic pathways in glioblastoma resected under and after bevacizumab treatment

**DOI:** 10.1007/s10014-024-00481-0

**Published:** 2024-04-15

**Authors:** Taketo Ezaki, Toshihide Tanaka, Ryota Tamura, Kentaro Ohara, Yohei Yamamoto, Jun Takei, Yukina Morimoto, Ryotaro Imai, Yuki Kuranai, Yasuharu Akasaki, Masahiro Toda, Yuichi Murayama, Keisuke Miyake, Hikaru Sasaki

**Affiliations:** 1 Department of Neurosurgery, Keio University School of Medicine, 35 Shinanomachi, Shinjuku-Ku, Tokyo, 160-8582 Japan; 2 Department of Neurosurgery, The Jikei University School, of Medicine Kashiwa Hospital, 163-1 Kashiwashita, Kashiwa-shi, Chiba, 277‐8567 Japan; 3 Department of Neurosurgery, The Jikei University School of Medicine, 3-25-8 Nishi-Shinbashi, Minato-Ku, Tokyo, 105-8461 Japan; 4 Department of Pathology, Keio University School of Medicine, 35 Shinanomachi, Shinjuku-Ku, Tokyo, 160-8582 Japan; 5 Department of Neurosurgery, The Jikei University School of Medicine Daisan Hospital, 4-11-1 Izumi-Motomachi, Komae-Shi, Tokyo, 201-8601 Japan; 6 Department of Neurosurgery, The Jikei University School of Medicine Katsushika Medical Center, 6-41-2 Aoto, Katsushika-Ku, Tokyo, 125-8506 Japan; 7 Department of Neurological Surgery, Faculty of medicine, Kagawa University Graduate School of Medicine, 1750-1 Miki-Choho, Ikenobe, Kita-Gun, Kagawa, 761-0793 Japan; 8 Department of Neurosurgery, Tokyo Dental College Ichikawa General Hospital, 5-11-13 Sugano, Ichikawa-Shi, Chiba, 272-8513 Japan

**Keywords:** Glioblastoma, Bevacizumab, Vascular endothelial growth factor, Alternative angiogenesis factor

## Abstract

Glioblastoma multiforme (GBM) acquires resistance to bevacizumab (Bev) treatment. Bev affects angiogenic factors other than vascular endothelial growth factor (VEGF), which are poorly understood. We investigated changes in angiogenic factors under and after Bev therapy, including angiopoietin-1 (ANGPT1), angiopoietin-2 (ANGPT2), placental growth factor (PLGF), fibroblast growth factor 2, and ephrin A2 (EphA2). Fifty-four GBM tissues, including 28 specimens from 14 cases as paired specimens from the same patient obtained in three settings: initial tumor resection (naïve Bev), tumors resected following Bev therapy (effective Bev), and recurrent tumors after Bev therapy (refractory Bev). Immunohistochemistry assessed their expressions in tumor vessels and its correlation with recurrent MRI patterns. PLGF expression was higher in the effective Bev group than in the naïve Bev group (*p* = 0.024) and remained high in the refractory Bev group. ANGPT2 and EphA2 expressions were higher in the refractory Bev group than in the naïve Bev group (*p* = 0.047 and 0.028, respectively). PLGF expression was higher in the refractory Bev group compared with the naïve Bev group for paired specimens (*p* = 0.036). PLGF was more abundant in T2 diffuse/circumscribe patterns (*p* = 0.046). This is the first study to evaluate angiogenic factors other than VEGF during effective and refractory Bev therapy in patient-derived specimens.

**Supplementary Information:**

The online version contains supplementary material available at 10.1007/s10014-024-00481-0.

## Introduction

In Japan, bevacizumab (Bev), a monoclonal antibody against the potent angiogenic factor, vascular endothelial growth factor (VEGF) has been approved for newly diagnosed and recurrent high-grade gliomas. Bev has become one of the standard therapeutic modalities for glioblastoma multiforme (GBM), especially for recurrence, but overall survival (OS) is not prolonged in the clinical trials of phase 3 study [1, 2]. Unfortunately, the effect of Bev treatment does not last, regardless of drastic regression of tumor volume.

To explore the mechanism for Bev resistance, we have conducted comparative analyses of VEGF expression levels, tumor oxygenation, stemness, and immunoregulatory mechanisms using samples from patients that were naïve to, treated with, and refractory to Bev treatment using paired samples obtained from initial and recurrent surgery [3–6]. In these studies, the microvessel density in the tumor microenvironment (TME) under Bev treatment was significantly decreased with improvement in tumor oxygenation, and in the majority of Bev-refractory samples, tumor hypoxia was recovered with a paradoxical decrease in microvessel density [5]. Reactivation of VEGF may not, therefore, be initially involved in the acquisition of resistance to Bev and alternative and salvage angiogenesis pathways other than those involving VEGF can be induced in Bev therapy resistance [7]. Given that VEGF plays a pivotal role in tumor angiogenesis in GBM, other angiogenic factors, including fibroblast growth factor 2 (FGF2; also known as basic fibroblast growth factor, bFGF) [7], placental growth factor (PLGF) [8], platelet-derived growth factor (PDGF) [9], angiopoietin-1 (ANGPT1) [10–13], angiopoietin-2 (ANGPT2) [14–17], and Ephrin A2 (EphA2) [18–20] are also involved. However, very few reports have explored multiple angiogenic pathways using paired tumor tissues from the same patients with newly diagnosed and recurrent GBM, particularly surgical samples obtained during Bev treatment [8, 9, 20, 21].

To understand the mechanism of Bev resistance and to overcome its transient therapeutic efficacy, involvement of angiogenic factors other than VEGF need to be investigated during Bev therapy.

We addressed the following questions:

1. How are angiogenic factors other than VEGF involved during effective and refractory of Bev therapy?
2. Are salvage angiogenic pathways activated after Bev failure?
3. There are two common patterns of magnetic resonance imaging (MRI) in recurrent GBM after Bev therapy, T1-contrast enhancement and non-enhancement. Do enhancement/non-enhancement MR images represent involvement of salvage angiogenesis pathways after failure of Bev therapy?

Comparative analyses between initial and salvage surgery by analysis of surgical samples can help clarify the mechanism of action and resistance of Bev therapy. This study aimed to investigate the status and changes of non-VEGF angiogenic factors during Bev therapy by analysis of surgical samples from initial and salvage surgery. This is the first comparative analysis of alternative angiogenesis pathways using human surgical specimens derived from patients during effective and resistance stages of Bev therapy.

## Patients and methods

### Patient eligibility and sample collection

**Fig. 1:**
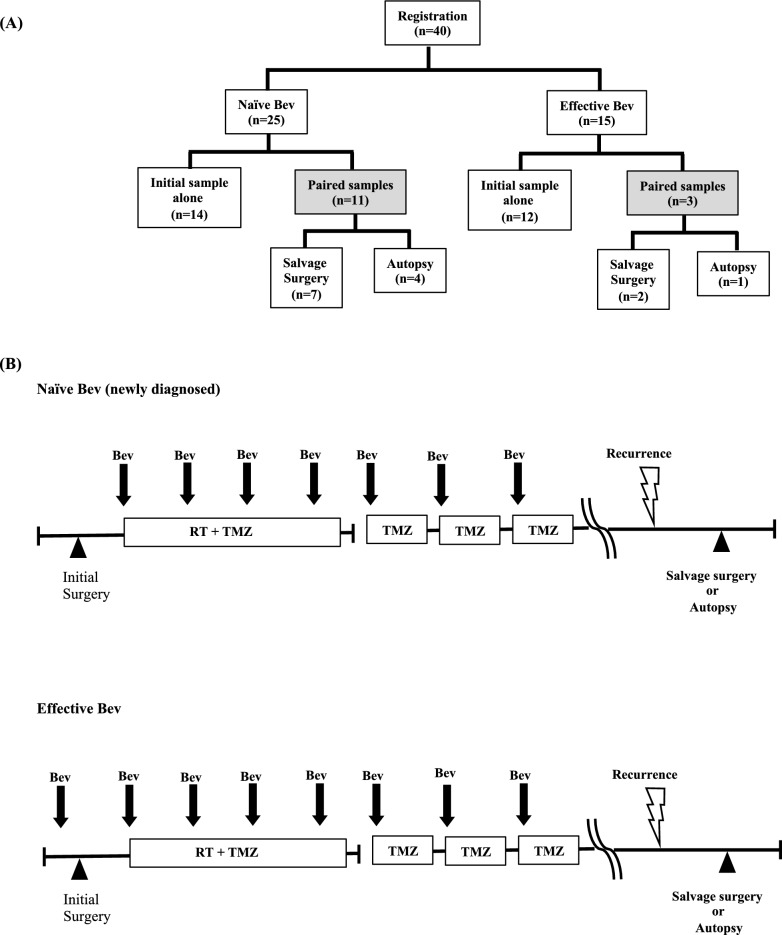
**A** Tissue sampling. Fifty-five tumor tissues obtained from three different settings: 15 tumor tissues were resected under neoadjuvant Bev (defined as “effective Bev”). Twenty-five tumor tissues were resected before Bev therapy (defined as “naïve Bev”). Fourteen tumor tissues were resected after Bev failure (defined as “refractory Bev”). Nine tumor tissues (seven from the naïve Bev group, and two from the effective Bev group) were obtained from salvage surgery. Five tumor tissues (four from the naïve Bev group, and one from the effective Bev group) were obtained from autopsy. **B** Treatment protocol of neoadjuvant Bev followed by surgery and postoperative adjuvant RT and TMZ combined with Bev. Surgery was performed in naïve, effective (just after preoperative neoadjuvant Bev), and refractory Bev periods. *Bev* bevacizumab, *RT* radiotherapy, *TMZ* temozolomide

The present study used 54 GBM tissues from 40 patients obtained from three different settings as previously described [4]; 15 tumors were removed after neoadjuvant Bev administration, i.e., during Bev response (effective Bev), 25 were newly diagnosed GBM without any previous treatment (naïve Bev), and 14 were recurrent tumors after Bev administration (refractory Bev). The refractory Bev group included five specimens by autopsy in addition to nine recurrent tumors obtained from salvage surgery (Fig. 1A). As paired specimens from the same patient, 22 naïve Bev-refractory Bev specimens from 11 cases (including four autopsy specimens and seven salvage surgery specimens) and six effective Bev-refractory Bev specimens from three cases (including one autopsy specimen and two salvage surgery specimens) were obtained.

### Treatment protocol

All effective Bev patients were treated with preoperative neoadjuvant bevacizumab (neoBev) at 10 mg/kg on day 0. Surgical resection was performed 3–4 weeks after neoBev administration. Maintenance treatment with TMZ began 4 weeks after the completion of radiotherapy at a starting dose of 150 mg/m^2^ for 5 consecutive days of a 28-day cycle. All newly diagnosed GBM patients (naïve Bev) were treated with concomitant Stupp regimen and Bev after surgical resection (Supplement Table 1) [1], (Fig. 1B).

### Study oversight

The protocol was approved by the ethics committee of Jikei University School of Medicine Kashiwa Hospital, Keio University School of Medicine, and Kagawa University Graduate School of Medicine. The study was approved by the Institutional Review Board (JKI18-052 for Jikei, N20160036 for Keio, 2018KH046 for Kagawa), and conducted in accordance with the Helsinki declaration. Written informed consent was obtained from all participants.

### Immunohistochemistry

To assess changes in expression levels of angiogenic factors other than VEGF during Bev therapy, ANGPT1, ANGPT2, FGF2, EphA2, and PLGF were analyzed using 4-μm sections of formalin-fixed, paraffin-embedded tissues. Procedures were followed according to manufacturers’ protocols. Briefly, antigen retrieval was performed by a microwave method in 10 mM citrate buffer (pH; 6.0). After blocking with 2.5% normal horse serum (ImmPress Detection Systems; Vectorlabs, Burlingame, CA, USA) for 60 min, the sections were incubated overnight at 4 °C with anti-ANGPT1 (1:200, bs-0800R, BIOSS, MA, USA), anti-ANGPT2 (1:200, ab155106, abcam, Cambridge, UK), anti-FGF2 (1:100, sc-74412, Santa Cruz, TX, USA), anti-EphA2 (1:100, NBP2-02810, Novus Biologicals, CO, USA) or anti-PLGF (1:100, ab19666, abcam, Cambridge, UK) antibodies [22]. Immunoreactivity was visualized by the peroxidase–diaminobenzidine reaction. Expression levels of the five angiogenic factors were assessed by immunohistochemistry in tumor vessels consisting of vascular endothelial cells, pericytes, and other cells such as infiltrating macrophage surround the vessel walls, and five representative hot spots were selected under high-power observational fields (HPFs) of 400×  magnification, 0.47 mm^3^. All experiments were assessed by five authors (TE, TT, RT, KO, and HS) and a consensus reached (Supplement Table 2).

### MRI at recurrence

All patients were followed up regularly with MRI. The recurrent MRI pattern was classified into two settings, cT1-flare up and T2 diffuse/circumscribed [23, 24].

### Statistical analyses

The Mann–Whitney *U* test was performed to statistically compare the levels of the angiogenic factors between any two groups. In paired samples, comparison was performed with a corresponding Wilcoxon signed ordered sum test. In addition, the Mann–Whitney *U* test was used to test association between the MRI pattern of recurrence and the expression of angiogenic factors.

## Results

### ANGPT1/ANGPT2

**Fig. 2:**
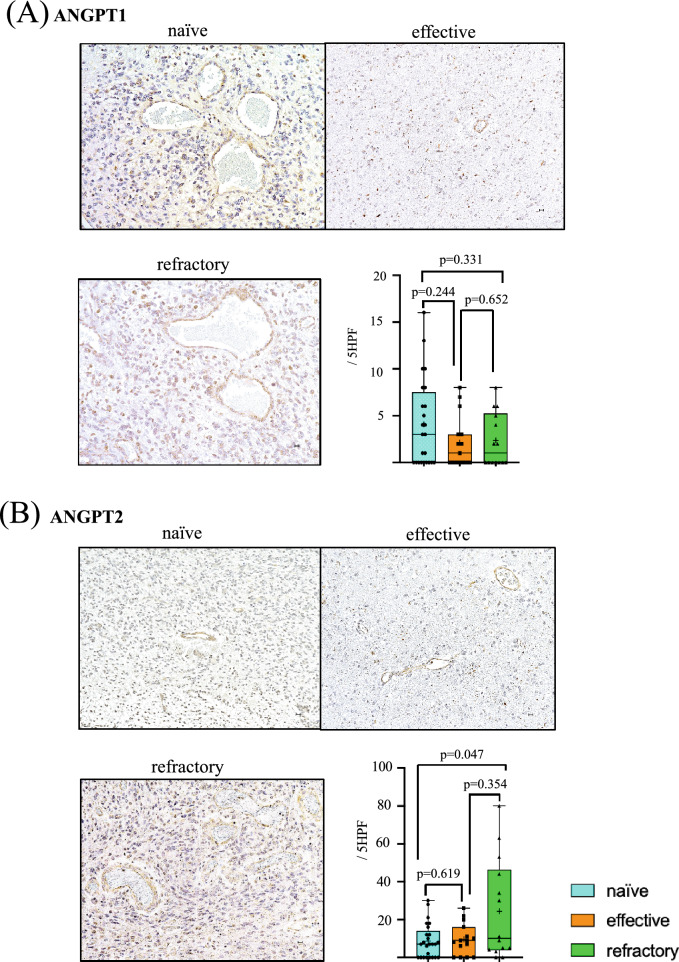

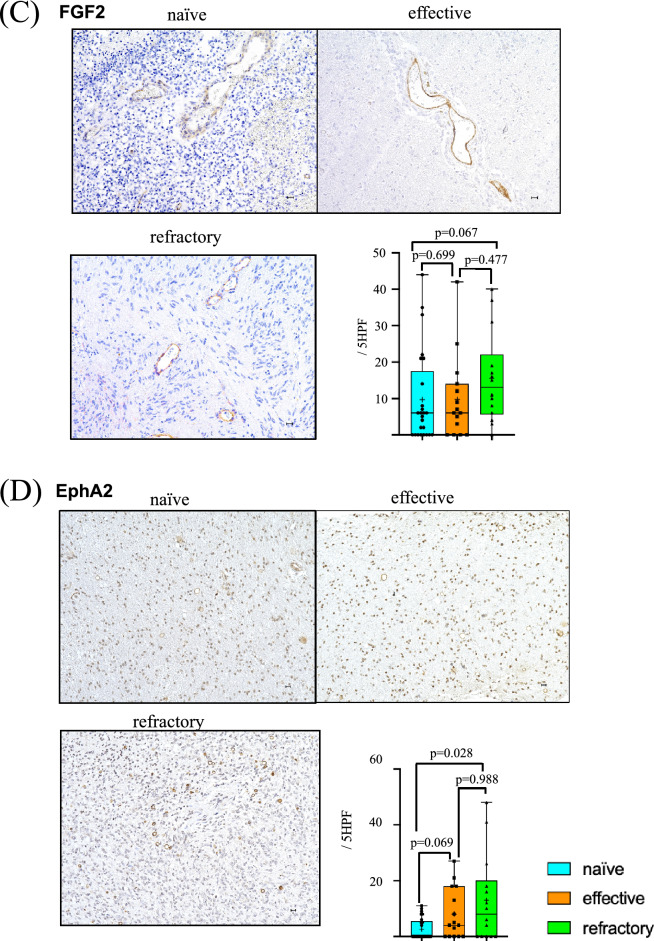

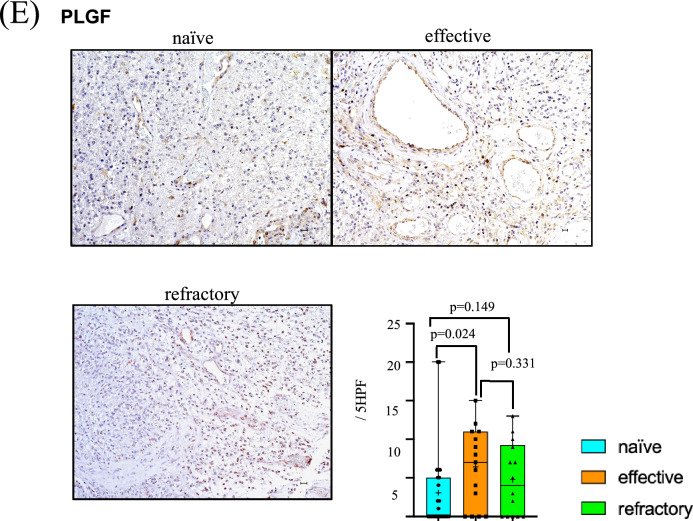
Levels of ANGPT1 (**A**), ANGPT2 (**B**), FGF2 (**C**), EphA2 (**D**) and PLGF (**E**) in tumor vessels. Immunohistochemistry photomicrographs shows naïve (upper left panel), effective (upper right panel) and refractory (bottom left panel). Numbers of positive vessels in tumor in five high power fields (5 HPF) were compared among naïve (blue), effective (orange), and refractory (green) Bev groups (bottom right panel)

Both expression of ANGPT1 and ANGPT2 were intensely present in vascular endothelial cells especially in larger sized tumor vessels (≥ 15 µm) and some small vessels (< 15 µm). There was no statistical difference in ANGPT1 expression in vascular endothelial cells among groups (naïve vs. effective; *p* = 0.244, effective vs. refractory; *p* = 0.652, naïve vs. refractory; *p* = 0.331) (Fig. 2A).

However, there was significantly robust expression of ANGPT2 in tumor vessels in the refractory Bev group (24.36/5 HPF) compared with that in the naïve (8.68/5 HPF) and effective (9.60/5 HPF) Bev groups. There was also a significant difference between naïve Bev and refractory Bev groups (*p* = 0.047) (Fig. 2B).

### FGF2

Compared with the other angiogenic factors, strong positive staining of FGF2 was detected in vascular endothelial cells especially in larger sized tumor vessels (≥ 15 µm). In contrast, there was very little staining in small tumor vessels (< 15 µm) (Fig. 2C). The expression level of FGF2 tended to increase from naïve (9.68/5 HPF) to effective (9.73/5 HPF) and refractory (15.50/5 HPF) Bev groups, although there was no significant difference.

### EphA2

In contrast to FGF2, distribution of EphA2 expression was mostly localized in small vessels (< 15 µm). Expression of EphA2 was elevated in the refractory Bev group (12.93/5 HPF) compared with the naïve Bev (2.64/5 HPF) and the effective Bev (8.07/5 HPF) groups. There was a statistical difference between expression levels in the naïve Bev and refractory Bev groups (*p* = 0.028) (Fig. 2D).

### PLGF

Distribution of PLGF expression was mostly localized in larger vessels (≥ 15 µm). Compared with tumor vessels in the naïve Bev group (3.08/5 HPF), expression level of PLGF was increased in vascular endothelial cells in effective (6.40/5 HPF) and refractory Bev (4.79/5 HPF) tumors. There was a statistical difference in the expression level of PLGF between effective Bev and naïve Bev groups (*p* = 0.024) (Fig. 2E).

### Comparative analyses using paired samples

**Fig. 3:**
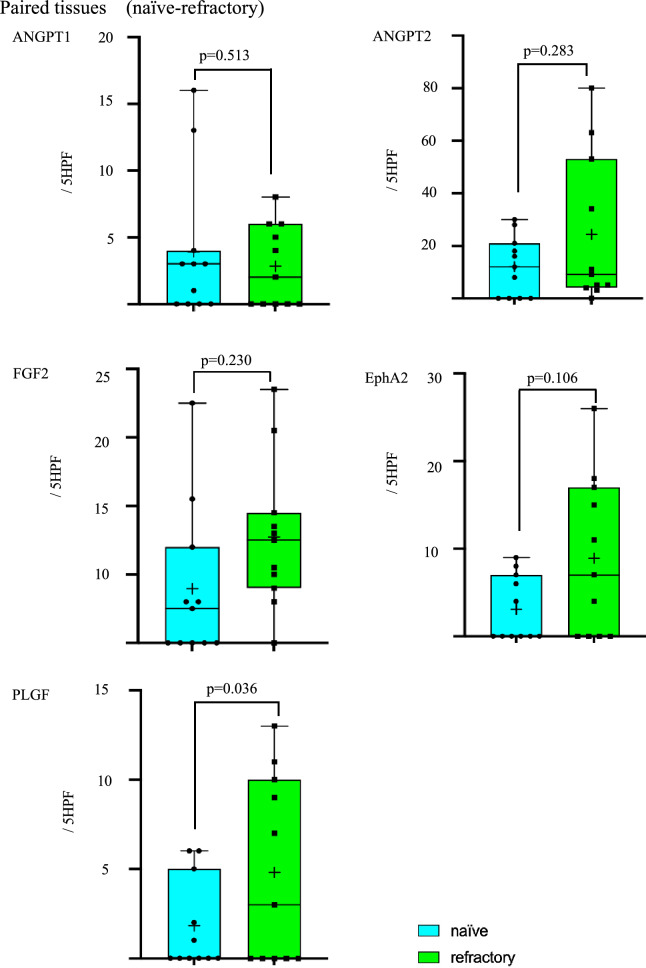
Paired comparisons of ANGPT1, ANGPT2, FGF2, EFNA2, and PLGF by immunoreactivity in tumor vessels between naïve (blue) and refractory (green) Bev groups. Numbers of positive vessels were quantitated from five HPFs

Comparison of paired samples obtained from naïve and refractory Bev patients showed expression levels of PLGF to be higher in refractory Bev compared with naïve Bev samples (*p* = 0.036). In contrast, there were no significance differences in expression levels of ANGPT1, ANGPT2, FGF2 or EphA2 between naïve and refractory Bev groups (Fig. 3). In addition, three paired samples obtained from effective and refractory Bev, there were no significance differences in the expression levels of any angiogenic factors (Supplement Fig. 1).

### Expression of angiogenic factors according to recurrent pattern on MRI

**Fig. 4:**
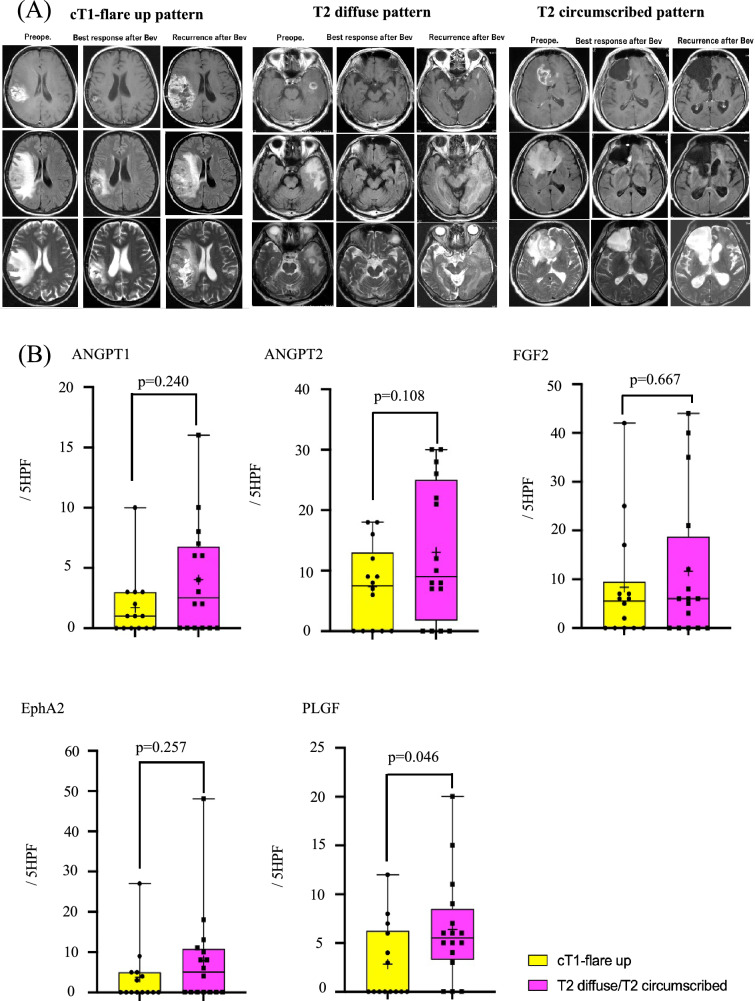
**A** Images of recurrent MRI (cT1-flare up and T2 diffuse/T2 circumscribed). Left panel shows cT1-flare up, middle panel shows T2 diffuse and right panel shows T2 circumscribed images. **B** Numbers of positive vessels of each recurrent pattern were quantitated in five HPFs. Yellow shows cT1-flare up and Pink shows T2-diffuse/T2 circumscribed pattern

Patterns of recurrence on MRI after Bev therapy were classified as previously described [5, 24] as cT1-flare up (*n* = 14) and T2 diffuse/circumscribed (*n* = 16) (Fig. 4A). In the refractory Bev group, expression levels of ANGPT1, ANGPT2, FGF2, EphA2 and PLGF in T2-diffuse/circumscribed tumors tended to be higher compared with levels in cT1-flare up tumors. In particular, there was statistically significant in the expression level of PLGF (*p* = 0.046) (Fig. 4B).

## Discussion

Preclinical studies of TMZ and Bev combination therapy for glioma demonstrated anti-tumor activity through inhibition of angiogenesis [25, 26]; however, clinical results were disappointing. Adaptation of the TME that leads to activation of redundant angiogenesis pathways is one mechanism that can lead to acquired resistance to anti-angiogenic therapies that target VEGF and its receptors [27]. Changes in angiogenic factors and cytokines have been described in patients treated with anti-angiogenic agents that target VEGF and tyrosine kinase [17, 28, 29]; however, few studies have analyzed paired samples from the same patients who underwent surgical resection during both naive and Bev-resistance stages [8, 9, 21]. In addition, it should be unique that we focused on expression of angiogenic factors other than VEGF as a salvage angiogenesis pathway in tumor vessel including vascular endothelial cell.

**Fig. 5:**
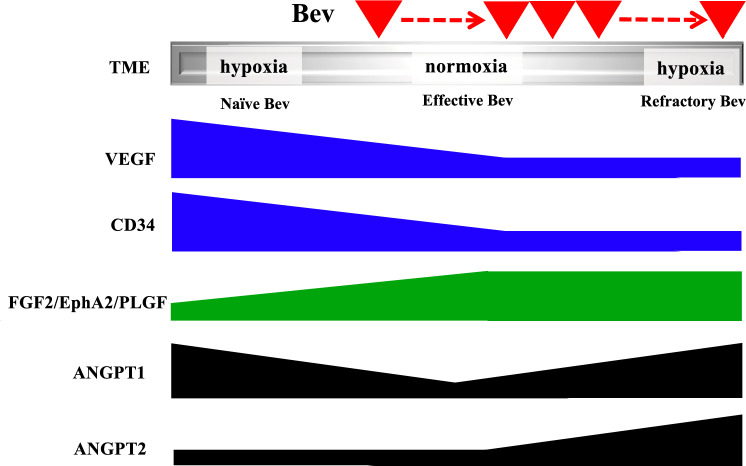
Scheme representing changes in VEGF, CD34, and alternative angiogenic factors in tumor vessels at naïve, effective and refractory Bev stages. FGF2, EphA2, and PLGF levels increase in the effective and refractory Bev stages. ANGPT1 tends to decrease in the effective Bev stage compared with the naïve Bev stage, and increase in the refractory Bev stage again, while ANGPT2 tends to increase in the refractory stage compared with naïve and effective Bev stages

Clinical trials of VEGF-targeted therapy for GBM have shown upregulated serum levels of ANGPT2, EphA2, and FGF2 in the refractory period [17, 20, 28], while changes in the expression level of PLGF were controversial [8, 10, 21, 29]. Here, we report that expressions of ANGPT2, EphA2, and PLGF are upregulated in tumors resistant to Bev therapy, while expression levels of ANGPT1 and FGF2 evaluated by immunohistochemistry remain stable (Fig. 2A, B). We previously showed that the TME becomes normoxic in the effective Bev stage and hypoxic in the refractory Bev stage, regardless of VEGF suppression [5]. This raises the question of whether changes in tumor oxygenation during VEGF-targeting therapy affect alternative angiogenesis pathways. The present study demonstrates changes in expression levels of angiogenic factors in heterogeneous TME during Bev therapy (Fig. 5).

FGF2, EphA2, and PLGF levels were upregulated in effective and refractory Bev stages under reduced vascular density (Fig. 2C, D, E) [5], regardless of therapeutic response. PLGF, belonging to the VEGF-family, was not altered between effective and refractory Bev stages [30]. PLGF was observed in tumor cells and vascular endothelial cells in hypoxic GBM, indicating that the TME under hypoxic conditions is also a source of PLGF [21]. PLGF elevation is significant during Bev therapy response in patients with metastatic colorectal cancer [31] and GBM [10], making it a reliable as a predictive biomarker in clinical outcomes.

Upregulation of EphA2 has potential as a novel immunotherapy for patients that are refractory to Bev [18–20, 32, 33]; however, the low expression level of EphA2 correlate with a favorable prognosis in the Cancer Genome Atlas GBM database [20], suggesting that EphA2 could also be a predictive biomarker and an alternative target for salvage therapy after Bev failure.

FGF2 is crucial for tumor angiogenesis and alternative angiogenic pathways during Bev therapy [7]. Multiple kinase inhibitors target VEGF, FGF, and PDGF receptor pathways to overcome resistance. Okamoto et al. investigated vascular structures and expression levels of angiogenic factors, including FGF2 and PDGF by paired comparison of initial surgery and autopsied samples after Bev failure [9]. Our present data demonstrated FGF2 to be upregulated at Bev therapy commencement, while a phase II study showed no significant change before or after Bev therapy, indicating no impact on clinical outcome [28, 34]. Further studies are needed to understand the significance of FGF2 induction in Bev therapy for GBM.

In naïve Bev, ANGPT1 facilitates vascular normalization and maturation during angiogenesis, whereas ANGPT2 has antagonist properties towards ANGPT1 [15, 35]. After VEGF blockade, expression level of ANGPT1 increased in a narrow therapeutic window during tumor oxygenation [13], while ANGPT2 induced vascular remodeling and sprouting under hypoxic TME at Bev resistance [14, 15, 36–38]. Our data show that ANGPT1 was downregulated in the effective Bev stage compared with the naïve Bev stage, while ANGPT2 remained unchanged until the refractory stage. Our previous and present data [5, 13, 16, 37, 38] indicate that ANGPT1 and ANGPT2 might be reciprocally regulated during tumor oxygenation but upregulated together in the refractory period (Fig. 5). It is also known that ANGPT2 compensates for VEGF inhibition by recruiting perivascular myeloid-derived suppressive cells and M2 macrophages [39, 40]. Upregulation of ANGPT2 is associated with T-cell exclusion, and blocking it promotes CD8+ T-cell infiltration, resulting in anti-tumor effects [41]. These evidences might support results of a clinical trial for bispecific antibodies targeting VEGF and ANGPT2 [42, 43], potentially supporting ANGPT2-targeted therapy combined with anti-VEGF therapy as a second-line therapy for patients with refractoriness of Bev. It also suggests that inhibition of ANGPT2 may overcome Bev resistance, and that combined immunotherapy may avoid tumor recurrence in hypoxic and immunosuppressive TME.

Radiographic comparison between enhancement and non-enhancement patterns in refractory Bev showed that there were no differences in expression levels of various angiogenic factors [23]. In the present study of recurrence patterns on MRI classified as T1-flare up and T2-diffuse pattern as previously described [5, 24], expression of all angiogenic factors examined in the present study was elevated in recurrent tumors with the T2-diffuse pattern on MRI. This discrepancy might be due to selection bias of autopsy samples as well as surgical resection of from non-enhancement pattern MRI tissue specimens in refractory Bev.

All angiogenic factors examined in the present study were upregulated in T2-diffuse/circumscribed pattern patients compared with cT1-flare up patients in pair cases of naïve-refractory, indicating that alternative angiogenesis pathways might be therapeutic targets when the non-enhancement pattern of recurrence on MRI occurs in patients with refractory to Bev.

In summary, this study reveals differential activation of salvage angiogenic pathways in surgical specimens, with FGF2, EphA2, and PLGF upregulated in tumor vessels, while ANGPT1 and ANGPT2 were downregulated and upregulated, respectively. Upregulation of angiogenic factors in hypoxic TME may compensate for a reduced blood supply, providing alternative therapeutic targets for recurrent GBM after VEGF-targeted therapy failure.

The study has several limitations. We assessed expression levels of angiogenic factors with focusing on tumor vessels including vascular endothelial cells, but it is difficult to detect expression levels of vascular endothelial cells accurately and strictly. It might be possible that pericytes and macrophage around tumor vessels were also included, consisting of tumor vessels. In addition, the present study was retrospective, limited to paired tissues from the same patients, and restricted to naïve and refractory Bev stages. The rarity of salvage surgery for recurrent GBM after Bev failure, RT, and TMZ makes achieving statistical significance difficult. In addition, paired samples were restricted to patients who underwent surgery for newly diagnosed GBM after preoperative neoadjuvant Bev [3]. Comparing paired samples between effective and refractory Bev stages is necessary to increase the study’s significance. Further studies using a larger number of patients are needed.

## Conclusion

This study reports the analysis of alternative angiogenic factor pathways (non-VEGF pathways) at naive, effective, and refractory stages using paired specimens. There was a divergence in levels at the time of response between FGF2/EphA2/PLGF and ANGPT1/2. By immunohistochemical analyses, expression levels of angiogenic factors other than VEGF were more elevated in the MRI non-enhancement pattern compared with the enhancement pattern.

### Supplementary Information

Below is the link to the electronic supplementary material.

Supplementary file1 (PDF 185 KB)

## Data Availability

The datasets generated during and/or analyzed during the current study are available from the corresponding author on reasonable request.
