# Correction: Status of alternative angiogenic pathways in glioblastoma resected under and after bevacizumab treatment

**DOI:** 10.1007/s10014-024-00485-w

**Published:** 2024-05-07

**Authors:** Taketo Ezaki, Toshihide Tanaka, Ryota Tamura, Kentaro Ohara, Yohei Yamamoto, Jun Takei, Yukina Morimoto, Ryotaro Imai, Yuki Kuranari, Yasuharu Akasaki, Masahiro Toda, Yuichi Murayama, Keisuke Miyake, Hikaru Sasaki

**Affiliations:** 1 Department of Neurosurgery, Keio University School of Medicine, 35 Shinanomachi, Shinjuku-Ku, Tokyo, 160-8582 Japan; 2 Department of Neurosurgery, The Jikei University School, of Medicine Kashiwa Hospital, 163-1 Kashiwashita, Kashiwa-shi, Chiba, 277‐8567 Japan; 3 Department of Neurosurgery, The Jikei University School of Medicine, 3-25-8 Nishi-Shinbashi, Minato-Ku, Tokyo, 105-8461 Japan; 4 Department of Pathology, Keio University School of Medicine, 35 Shinanomachi, Shinjuku-Ku, Tokyo, 160-8582 Japan; 5 Department of Neurosurgery, The Jikei University School of Medicine Daisan Hospital, 4-11-1 Izumi-Motomachi, Komae-Shi, Tokyo, 201-8601 Japan; 6 Department of Neurosurgery, The Jikei University School of Medicine Katsushika Medical Center, 6-41-2 Aoto, Katsushika-Ku, Tokyo, 125-8506 Japan; 7 Department of Neurological Surgery, Faculty of medicine, Kagawa University Graduate School of Medicine, 1750-1 Miki-Choho, Ikenobe, Kita-Gun, Kagawa, 761-0793 Japan; 8 Department of Neurosurgery, Tokyo Dental College Ichikawa General Hospital, 5-11-13 Sugano, Ichikawa-Shi, Chiba, 272-8513 Japan

**Correction: Brain Tumor Pathology (2024) 41:61–72** 10.1007/s10014-024-00481-0

In the original publication of the article, the author name Yuki Kuranari was incorrectly written as Yuki Kuranai.

