# Supplementary material for: Status of alternative angiogenic pathways in glioblastoma resected under and after bevacizumab treatment
Source: Brain Tumor Pathol. 2024 Apr 15;41(2):61–72. doi: 10.1007/s10014-024-00481-0 (PMC11052834; doi:10.1007/s10014-024-00481-0)
Supplement: Supplementary file 1 — Supplementary file1 (PDF 185 KB) [file 10014_2024_481_MOESM1_ESM.pdf]

Supplementary Information

article title: Status of alternative angiogenic pathways in glioblastoma resected under and after bevacizumab treatment

journal name: Brain Tumor Pathology

author names: Taketo Ezaki

affiliation and e-mail address of the corresponding author:
Toshihide Tanaka, MD, PhD
Department of Neurosurgery, Jikei University School of Medicine
3-25-8 Nishi-Shinbashi, Minato-ku, Tokyo 105-8461, Japan
TEL: +81-3-3433-1111 (ext. 3461); FAX: +81-3-3459-6412
E-mail: ttanaka@jikei.ac.jp

Supplement Table1

Characteristics of patients stratified by responsiveness of Bev

Characteristic	timing for obtaining of samples during Bev therapy		
	naïve Bev (n=25)	effective Bev (n=15)	refractory Bev (n=14)
**Age: Median (years old) [range],yr**	55 [29-79]	68 [48-80]	52 [29-77]
**Male (n)**	18	9	8
**Female (n)**	7	6	6
**no. cycles of neo-adjuvant Bev**	0	1 Time, 11<br>2 Times, 1<br>3 Times, 3	
**Interval between last Bev to surgery/autopsy (days) [range]**		23 [15-51]	33 [5-208]
**no. cycles of Bev: median [range]**	3 [1-24]	9 [0-28]	
**no. cycles of TMZ: median [range]**	12 [1-28]	8 [1-24]	
***IDH-1* wild status: (n)**	23	14	
**Methylation status of promoter region of *MGMT* (n)**	6	2	
**MRI pattern of recurrence**			
**cT1-Flare up (n)**	6	8	
**T2 diffuse / T2 circumscribed (n)**	10	6	
**non-responder (n)**	4	0	

no: Number
Bev: Bevacizumab
TMZ: Temozolomide
***IDH:* isocitrate dehydrogenase**
***MGMT*: O6-methylguanine–DNA methyltransferase**

Supplement Table2

Characteristics of genetic status, content of chemotherapy, and number of angiogenic factors positive cells

	Case	age/sex	genetic status		no. cycles of Bev/TMZ			MRI pattern of recurrence	number of angiogeneic factors positive cell in tumor vessels				
			IDH-1 status	*MGMT* status	no. cycles of neo-adjuvant Bev	no. cycles of Bev	no. cycles of TMZ		ANGPT1	ANGPT2	FGF2	EFNA2	PLGF
**Naïve Bev**	N1<br>N1-Rs	29/F	WT	N/A		19	3	non-responder	3<br>0	0<br>4	0<br>15	2<br>0	0<br>0
	N2<br>N2-Rs	67/M	WT	N/A		21	19	T2 circumscribed	0<br>0	0<br>0	0<br>11	6<br>11	11<br>18
	N3<br>N3-Rs	51/F	WT	N/A		18	16	cT1 flare-up	1<br>4	0<br>63	0<br>31	0<br>0	0<br>0
	N4<br>N4-Rs	50/F	WT	N/A		14	20	non-responder	13<br>2	16<br>11	14<br>17	0<br>13	8<br>26
	N5<br>N5-Rs	52/F	WT	N/A		9	16	T2 diffuse	16<br>8	30<br>80	0<br>10	0<br>3	0<br>0
	N6<br>N6-Rs	73/M	WT	N/A		13	26	T2 diffuse	0<br>6	12<br>34	6<br>19	5<br>10	0<br>4
	N7<br>N7-Rs	52/M	WT	N/A		20	41	T2 diffuse	3<br>5	28<br>53	35<br>16	0<br>9	8<br>6
	N8<br>N8-Rs	33/F	WT	N/A		27	4	non-responder	0<br>0	8<br>3	0<br>37	5<br>2	10<br>0
	N9<br>N9-Rs	41/M	WT	N/A		26	26	T2 diffuse	4<br>0	21<br>9	21<br>6	1<br>7	4<br>16
	N10<br>N10-Rs	51/M	WT	N/A		9	15	T2 circumscribed	0<br>6	0<br>5	5<br>8	6<br>0	0<br>12
	N11<br>N11-Rs	56/M	WT	N/A		28	21	cT1 flare-up	3<br>0	18<br>5	6<br>3	0<br>0	0<br>10
	N12	36/M	R132H mut	Met		0	7	non-responder	0	2	0	0	0
	N13	51/M	WT	Met		2	2	N/A	0	0	4	0	5
	N14	77/M	WT	Met		3	13	N/A	5	0	33	0	0
	N15	44/M	WT	Unmet		19	6	T2 circumscribed	8	7	6	0	0
	N16	72/M	WT	Unmet		0	11	cT1 flare-up	10	12	7	6	5
	N17	57/M	WT	Met		0	0	cT1 flare-up	0	18	6	0	9
	N18	64/F	WT	Unmet		0	11	cT1 flare-up	1	0	0	0	0
	N19	55/M	WT	Unmet		N/A	14	T2 diffuse	10	8	44	2	0
	N20	66/M	WT	Unmet		0	5	T2 circumscribed	6	7	8	20	6
	N21	79/M	WT	Met		0	12	N/A	6	7	21	0	0
	N22	59/M	WT	Unmet		0	1	cT1 flare-up	0	0	2	4	0
	N23	48/M	WT	Unmet		11	11	N/A	0	7	2	0	0
	N24	56/M	WT	Met		0	6	N/A	8	10	22	20	0
	N25	71/F	WT	Unmet		0	14	N/A	4	6	0	0	0
	Case	age/sex	genetic status		no. cycles of Bev/TMZ			MRI pattern of recurrence	number of angiogeneic factors positive cell in tumor vessels				
			IDH-1 status	*MGMT* status	no. cycles of neo-adjuvant Bev	no. cycles of Bev	no. cycles of TMZ		Ang1	Ang2	bFGF	EphrinA2	PlGF
**Effective Bev**	E1	48/M	R132H mut	Met	3	3	3	cT1 flare-up	1	7	0	12	0
	E2<br>E2-Rs	51/M	WT	Unmet	2	2	2	cT1 flare-up	0<br>0	11<br>44	9<br>4	9<br>0	21<br>41
	E3<br>E3-Rs	55/M	WT	N/A	1	18	38	T2 diffuse	8<br>2	20<br>0	14<br>0	10<br>7	4<br>0
	E4	68/M	WT	N/A	1	1	0	T2 diffuse	6	22	12	15	8
	E5<br>E5-Rs	77/F	WT	N/A	1	9	17	T2 circumscribed	0<br>0	0<br>30	6<br>40	11<br>5	18<br>48
	E6	68/M	WT	N/A	1	24	35	cT1 flare-up	0	0	6	11	18
	E7	53/F	WT	N/A	1	9	24	T2 diffuse	2	6	5	0	0
	E8	72/M	WT	N/A	1	2	4	cT1 flare-up	7	26	3	4	53
	E9	72/M	WT	N/A	1	1	1	cT1 flare-up	0	0	0	7	5
	E10	61/F	WT	Met	3	3	3	cT1 flare-up	0	9	7	0	27
	E11	76/F	WT	Unmet	3	3	3	cT1 flare-up	0	16	25	0	3
	E12	80/F	WT	N/A	1	8	24	T2 diffuse	2	10	0	6	0
	E13	58/F	WT	Unmet	1	3	8	cT1-flare-up	3	9	42	3	4
	E14	69/M	WT	Unmet	1	12	8	cT1 flare-up	3	8	17	8	0
	E15	50/M	WT	N/A	1	8	12	cT1 flare-up	0	0	0	0	0

ANGPT; angiopoietin, Bev; bevacizumab, E; effective Bev, EFNA2; Ephrin A2, F; female, FGF2; basic fibroblast growth factor,, IDH; isocitrate dehydrogenase, M; male,
Met: Methylation of promoter region of MGMT, MGMT; methyl guanine methyltransferase, N; naïve Bev, N/A; not analyzed, PLGF; placenta growth factor,
Ra; sample in refractory Bev obtained from autopsy, Rs; sample in refractory Bev obtained from salvage surgery, TMZ; temozolomide,
Unmet; Unmethylation of promoter region of MGMT, WT; wild type
